# Supplementary material for: The Systems Biology Research Tool: evolvable open-source software
Source: BMC Syst Biol. 2008 Jun 29;2:55. doi: 10.1186/1752-0509-2-55 (PMC2446383; doi:10.1186/1752-0509-2-55)
Supplement: Additional file 1 — SBRT Archive. An archive of the current version of the Systems Biology Research Tool. [file 1752-0509-2-55-S1.zip › sbrt-1.4.0/doc/developers_guide/api/sbrt/shell/ProgramSolverFactory.html]

ProgramSolverFactory


|  |  |  |
| --- | --- | --- |
| **Overview** \| **Package** \| **Class** \| **Use** \| **Tree** \| **Deprecated** \| **Index** \| **Help** | |  |
| **PREV CLASS**   NEXT CLASS | **FRAMES**    **NO FRAMES**     **All Classes** |  |
| SUMMARY: NESTED \| FIELD \| CONSTR \| METHOD | DETAIL: FIELD \| CONSTR \| METHOD |  |


---


## sbrt.shell Class ProgramSolverFactory

```
java.lang.Object
  sbrt.shell.ProgramSolverFactory
```

---

``` public class ProgramSolverFactory extends java.lang.Object ```

This class is used to create `ProgramSolver` objects.

**Author:**
:   This class was written and documented by Jeremiah Wright while in the
    Wagner lab.

---

| **Field Summary** | |
| --- | --- |
| `static java.lang.String` | `CPLEX`             The string used to denote the CPLEX program solver from ILOG. |
| `static java.lang.String` | `GLPK`             The string used to denote the GLPK program solver from GNU. |
| `static UnmodifiableMap<java.lang.String,java.lang.String>` | `nameClassMap`             The correspondence between the strings used to denote a particular program solver and the name of the actual class it represents. |
| `static java.lang.String` | `PARAM_FILE`             The keyword used to denote a parameter file for a program solver. |
| `static java.lang.String` | `SOLVER`             The keyword used to denote the name of a program solver. |


| **Method Summary** | |
| --- | --- |
| `static ApplicationException` | `interpret(ProgramSolverException e)`             Interprets a program solver exception and returns a corresponding application exception. |
| `static <R,C> ProgramSolver<R,C>` | `make(ManagerInput<java.lang.String,java.lang.String> input, java.io.PrintWriter out)`             Uses the information contained in the provided input to create a new program solver object. |

**Methods inherited from class java.lang.Object**

`clone, equals, finalize, getClass, hashCode, notify, notifyAll, toString, wait, wait, wait`

**Field Detail**

### SOLVER

```
public static final java.lang.String SOLVER
```

:   The keyword used to denote the name of a program solver.

    **See Also:**: Constant Field Values

---


### PARAM\_FILE

```
public static final java.lang.String PARAM_FILE
```

:   The keyword used to denote a parameter file for a program solver.

    **See Also:**: Constant Field Values

---


### GLPK

```
public static final java.lang.String GLPK
```

:   The string used to denote the GLPK program solver from GNU.

    **See Also:**: Constant Field Values

---


### CPLEX

```
public static final java.lang.String CPLEX
```

:   The string used to denote the CPLEX program solver from ILOG.

    **See Also:**: Constant Field Values

---


### nameClassMap

```
public static final UnmodifiableMap<java.lang.String,java.lang.String> nameClassMap
```

:   The correspondence between the strings used to denote a particular
    program solver and the name of the actual class it represents. The keys
    of this map are the strings used to denote a program solver and the
    values of this map are the names of the actual class it represents.


**Method Detail**

### make

```
public static <R,C> ProgramSolver<R,C> make(ManagerInput<java.lang.String,java.lang.String> input,
                                            java.io.PrintWriter out)
```

:   Uses the information contained in the provided input to create a new
    program solver object. The keyword {#link `SOLVER` is required to
    be present in the input, and the keyword `PARAM_FILE` is optional.
    If `PARAM_FILE` *is* present, the program solver indicated by
    `SOLVER` must have a functioning
    `ProgramSolver.setParamFile(String)` method.

    :   **Type Parameters:**: `R` - the row type.: `C` - the column type. **Parameters:**: `input` - the input from which information is used to create a new program solver object.: `out` - the print writer to which status messages will be printed. **Returns:**: the program solver corresponding to the provided input. **Throws:**: `ApplicationException` - if a problem ocurrs while creating a program solver object.: `java.lang.UnsupportedOperationException` - if a call to `ProgramSolver.setParamFile(String)` fails.

---


### interpret

```
public static ApplicationException interpret(ProgramSolverException e)
```

:   Interprets a program solver exception and returns a corresponding
    application exception.

    :   **Parameters:**: `e` - the program solver exception to be interpretted. **Returns:**: an application exception that is the intrpretted version of the provided program solver exception.


---


|  |  |  |
| --- | --- | --- |
| **Overview** \| **Package** \| **Class** \| **Use** \| **Tree** \| **Deprecated** \| **Index** \| **Help** | |  |
| **PREV CLASS**   NEXT CLASS | **FRAMES**    **NO FRAMES**     **All Classes** |  |
| SUMMARY: NESTED \| FIELD \| CONSTR \| METHOD | DETAIL: FIELD \| CONSTR \| METHOD |  |


---
